# Investigating asymmetric salt profiles for nanopore DNA sequencing with biological porin MspA

**DOI:** 10.1371/journal.pone.0181599

**Published:** 2017-07-27

**Authors:** Ian C. Nova, Ian M. Derrington, Jonathan M. Craig, Matthew T. Noakes, Benjamin I. Tickman, Kenji Doering, Hugh Higinbotham, Andrew H. Laszlo, Jens H. Gundlach

**Affiliations:** Department of Physics, University of Washington, Seattle, WA, United States of America; Northeastern University, UNITED STATES

## Abstract

Nanopore DNA sequencing is a promising single-molecule analysis technology. This technique relies on a DNA motor enzyme to control movement of DNA precisely through a nanopore. Specific experimental buffer conditions are required based on the preferred operating conditions of the DNA motor enzyme. While many DNA motor enzymes typically operate in salt concentrations under 100 mM, salt concentration simultaneously affects signal and noise magnitude as well as DNA capture rate in nanopore sequencing, limiting standard experimental conditions to salt concentrations greater than ~100 mM in order to maintain adequate resolution and experimental throughput. We evaluated the signal contribution from ions on both sides of the membrane (*cis* and *trans*) by varying *cis* and *trans* [KCl] independently during phi29 DNA Polymerase-controlled translocation of DNA through the biological porin MspA. Our studies reveal that during DNA translocation, the negatively charged DNA increases cation selectivity through MspA with the majority of current produced by the flow of K^+^ ions from *trans* to *cis*. Varying *trans* [K^+^] has dramatic effects on the signal magnitude, whereas changing *cis* [Cl^-^] produces only small effects. Good signal-to-noise can be maintained with *cis* [Cl^-^] as small as 20 mM, if the concentration of KCl on the *trans* side is kept high. These results demonstrate the potential of using salt-sensitive motor enzymes (helicases, polymerases, recombinases) in nanopore systems and offer a guide for selecting buffer conditions in future experiments to simultaneously optimize signal, throughput, and enzyme activity.

## Introduction

**Fig 1:**
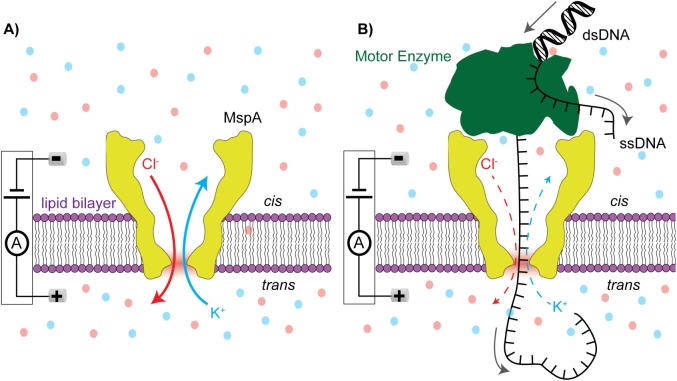
Basic nanopore sensing and sequencing schematic. (A) A single MspA channel is inserted into a lipid bilayer membrane. Positive (blue) K^+^ ions and negative (red) Cl^-^ ions are contained on either side of the membrane. An applied electric field drives K^+^ ions from the *trans* chamber to the *cis* chamber and Cl^-^ ions from *cis* to *trans* through MspA, producing the unblocked pore current. The region shaded in red at the base of the pore marks the constriction zone of MspA. (B) A motor enzyme controls the translocation of single stranded DNA through MspA. In the schematic depicted here, the motor enzyme translocates and unwinds double stranded DNA (dsDNA), allowing the passage of the ssDNA further into the pore with each step. The flow of both K^+^ and Cl^-^ ions is modulated by the presence of the DNA within the pore.

Nanopore DNA sequencing is an intrinsically fast single-molecule DNA sequencing technology that offers long read length at low costs [1, 2]. In canonical nanopore sequencing (Fig 1), a voltage is applied across a lipid membrane containing a single biological pore protein. Positive and negative ions contained in the salt chambers on either side of the membrane (*cis* and *trans*) are driven through the pore by the membrane electric field (Fig 1A), producing a measurable ion current. Negatively charged DNA molecules in the *cis* chamber will be attracted to and translocate through the pore towards the positive *trans* chamber. DNA within the pore affects the flow of ions, changing the measured ion current. Because the blockage currents depend on the specific DNA nucleotides passing through the pore, these signals can be used to identify DNA strand sequence [3, 4]. Biological nanopore Mycobacterium smegmatis porin A (MspA) has favorable physical dimensions for nanopore sequencing. Specifically, MspA’s small constriction zone of 1.2 nm diameter by 0.6 nm length (shown in shaded red in Fig 1A and Fig 1B), produces well resolved ion discrepancies between the four DNA nucleotides [3, 5]. However, single-stranded DNA translocates through the nanopore too quickly to measure the nucleotide-specific ion currents [5]. To realize nanopore DNA sequencing, a DNA motor enzyme is used to slow DNA translocation to the rate at which the enzyme processes the DNA strand, enabling sufficient time averaging of the ion currents as each nucleotide passes through the constriction of MspA (Fig 1B). In 2012, phi29 DNA polymerase (DNAP) was shown to ratchet DNA successfully through MspA in single-nucleotide steps with resolvable durations [4, 6].

Although, phi29 DNAP has proven effective for controlling DNA translocation through MspA, many DNA motor enzymes exist that could fulfill the role of phi29 DNAP in nanopore sequencing, including other polymerases, helicases, and translocases [7]. Enzymes with different traits, including increased processivity and stepping regularity, may improve sequencing accuracy and throughput. Testing new enzymes requires matching *cis* buffer conditions (salt concentration, pH, temperature) to the range of enzyme operating conditions. Conditions vary broadly depending on the intracellular conditions of the host organism that produced the enzyme. Even though both lipid bilayers and MspA are robust to a wide range of conditions, reducing salt concentrations for salt-sensitive enzymes can be problematic in nanopore sequencing. Salt concentration is intrinsically linked to nanopore ion current readings. With *cis* and *trans* buffer concentrations below ~100 mM KCl, signal-to-noise ratios in ion current measurements are significantly reduced. As a result, the changes in ion current resulting from the changing nucleotide positioning in the pore during DNA translocation become difficult to distinguish. Additionally, buffer salt concentration can influence the rate of DNA capture and threading into the nanopore, affecting the initial reagent concentrations ([DNA], [enzyme]) required for experimental throughput. For instance, using biological porin *α* Hemolysin, DNA capture rate was reduced over an order of magnitude between 0.5 M and 0.3 M KCl [8].

While the motor enzyme requires specific *cis* well buffer conditions to operate, the impermeable lipid membrane separating *cis* and *trans* allows for independent ion concentration in the *trans* chamber. Asymmetric salt profiles, *i*.*e*. different *cis* and *trans* ion concentrations, have been used previously in nanopore systems. DNA capture rate, DNA translocation dwell time, and DNA specific ion currents were shown to be modulated using salt gradients [9–12]. We hypothesized that using asymmetric salt profiles could maintain signal-to-noise ratio (SNR) with a low *cis* ion concentration by maximizing the ion concentration in *trans*. Assessing the viability of this technique requires determining the relative contribution of the ions in *cis* and *trans* to the ion currents through the nanopore.

Here, we investigated the influence of positive ion (K^+^) flow (*trans* to *cis*) and negative ion (Cl^-^) flow (*cis* to *trans*) on the signal magnitude and noise in ion currents measured through MspA. At many asymmetric salt profiles, we measured both unblocked pore ion currents and phi29 DNAP controlled DNA translocation ion currents through MspA under an applied electric field. In addition, we measured the effect of these salt gradients on free DNA capture rate and compare these results to previous studies.

## Materials and methods

### Membrane and pore establishment

Our experimental setup was previously described [4]. Phospholipid bilayers were composed of a mixture of a 1,2-diphytanoyl-sn-glycerol-3-phosphocholine (DPhPC from Avanti Polar Lipids) and hexadecane. DPhPC phospholipids have a zwitterionic headgroup, which prevents unbalanced ion buildup along the membrane surface. MspA mutant M2-NNN, with negative charges removed from the constriction [5], was used as the nanopore in all experiments. For simplicity, the M2-NNN mutant is referred to as MspA throughout the text. Single MspA insertions were isolated with the final *trans* solution in both *cis* and *trans*. After pore isolation, 1 ml of the final *cis* solution was perfused into the *cis* chamber (volume = ~60 μl).

### Operating conditions

#### DNA translocation experiments with phi29 DNAP

For varying *cis* well experiments, final *cis* conditions were [varying] KCl with 10 mM HEPES at pH 8.00 ± 0.05, 5 nM DNA, 500 nM phi29 DNAP, 1 mM dNTPs, 10 mM MgCl_2_, and 1 mM dithiothreitol (DTT). *Cis* [Cl^-^] is indicated in results instead of [KCl] as the electric field direction drives Cl^-^ ions from *cis* to *trans*. *Cis* [Cl^-^] is equal to *cis* [KCl] plus 20 mM from MgCl_2_ addition. Final *trans* conditions were 500 mM KCl with 10 mM HEPES at pH 8.00 ± 0.05.

For varying *trans* well experiments, final *cis* conditions were 200 mM KCl with 10 mM HEPES at pH 8.00 ± 0.05, 5 nM DNA, 500 nM phi29 DNAP, 1 mM dNTPs, 10 mM MgCl_2_, and 1 mM DTT. Final *trans* conditions were [varying] KCl with 10 mM HEPES at pH 8.00 ± 0.05. *Trans* [K^+^] is indicated in results instead of [KCl] as the electric field direction drives K^+^ ions from *trans* to *cis*. *Trans* [K^+^] is equal to *trans* [KCl] plus 8 mM from KOH buffering.

#### DNA Capture Rate experiments

*Cis* well conditions were [indicated] KCl, with 10 mM HEPES at pH 8.00 ± .05, and 500 nM DNA. T*rans* well conditions were KCl [indicated] with 10mM HEPES at pH 8.00 ± 0.05.

All experiments were performed at room temperature (21°C).

### Proteins

M2-NNN-MspA (accession number: CAB56052.1), phi29 DNAP (accession number: P03680.1)

### DNA construct design

For phi29 DNAP—DNA translocation experiments, DNA constructs were assembled with a 1:1:1.2 ratio of DNA template: primer: blocking oligo to a final concentration of 50 μM. DNA was annealed by heating to 95°C for 5 min, cooling to 60° C for 2 min, and then cooling to 4°C. The DNA template strand contains an extended 5’ end with a phosphate group to enhance threading into the nanopore. The primer contains a cholesterol on the 5’ end to associate with the bilayer and enhance capture rate. During each phi29 DNAP—DNA translocation event, the DNA strand is passed through the pore twice, once 5’ to 3’ (unzipping mode) and once 3’ to 5’ (synthesis mode) This method has been described in detail previously [4, 13].

For DNA Capture Rate experiments, DNA template and hairpin primer were mixed at a 1:1 ratio and annealed as above. The DNA constructs do not contain a cholesterol adapter to avoid the effect of changing bilayer sizes and densities on capture rate measurements with cholesterol tagged DNA. Detailed construct schematics and sequences are shown in S1 Table and S1 Fig.

### Data acquisition and analysis

All ion currents were recorded at 50 kHz on an Axopatch 200B amplifier with custom Labview Software (National Instruments) at an applied voltage of 180 mV and downsampled to 5 kHz. Ag/AgCl reference electrodes were used to apply voltages and to measure ion currents. Ag/AgCl electrodes were in direct contact with *cis* and *trans* solutions (no salt bridge). Because electrode potential is a function of the surrounding [Cl^-^], in asymmetric conditions (different *trans* and *cis* [Cl^-^]), the two electrodes have different electrode potentials, manifesting in an offset voltage. In each experiment, after isolating a single pore and reaching the desired asymmetric salt profile, an offset ion current was measured through the open pore at 0 mV applied voltage due solely to the offset voltage. In order to account for this effect, using the rezero function on the Axopatch 200B amplifier, the offset ion current was minimized, applying a counter offset voltage equal and opposite to the offset voltage. This ensures that no additional voltage was being applied to the system due to varying [KCl] in *cis* and *trans*. This counter offset voltage was monitored and maintained throughout the course of each experiment.

**Fig 2:**
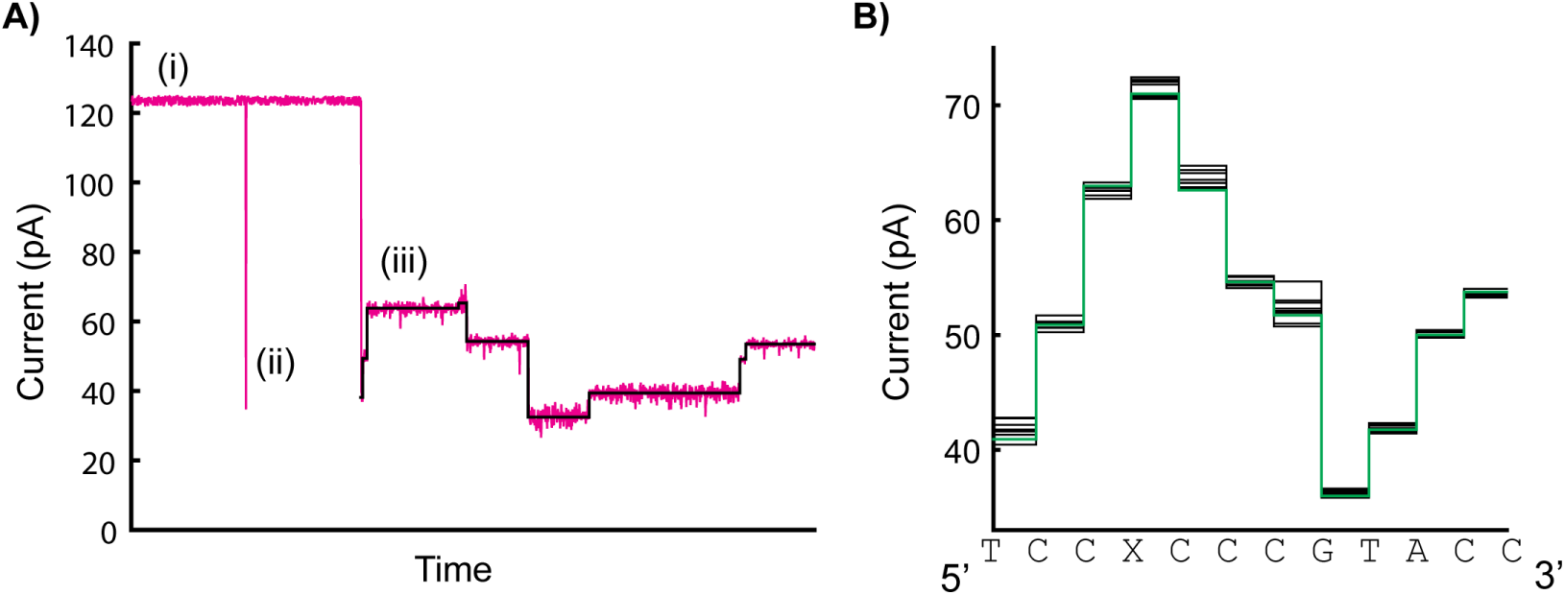
Example raw data trace and post-processed data traces. (A) The ion flow through MspA is recorded by measuring the current over time. (i) The unblocked pore produces a stable and reproducible current. (ii) Without the presence of a motor enzyme, DNA will translocate through MspA from *cis* to *trans* and reduce the measured current, but translocation occurs too quickly to resolve current changes produced by specific nucleotides in the DNA. (iii) With motor enzyme present, controlling DNA motion in the pore, discrete changes in current are observable with each step of the enzyme. A level finding algorithm identifies these discrete current states (levels) during controlled DNA translocation. (B) The DNA sequence present in the constriction of MspA during each translocation step determines the current pattern. After recording many controlled translocation events of the same DNA sequence and extracting the corresponding current levels, duration information resulting from stochastic enzymatic stepping behavior is removed. The individual current level patterns (black lines in (B)) are aligned and used to generate a consensus plot (green line in (B)) by taking the mean value of all the individual levels corresponding to each step. The DNA sequence producing the consensus current pattern is plotted. For each DNA translocation step, ~4 nucleotides present in the constriction zone of MspA affect the current magnitude [4]. ‘X’ denotes an abasic residue.

Our data analysis methods were previously described [7, 14]. Briefly, unblocked pore ion current was first determined by finding the peak in the histogram of all the measured ion currents. DNA translocation events were determined using a thresholding method on ion current data. For each DNA translocation event, a level finding algorithm was used to determine all of the discrete ion current levels and transitions [14] (Fig 2A). Consensus ion current plots for each asymmetric salt condition were generated using custom software [7] (Fig 2B). We used data from both phi29 unzipping and synthesis translocation modes in generating the consensus plots. For SNR calculations, a student t-test was used for each level transition, equal to the absolute value of the difference in median currents divided by the quadrature sum of their noise measurements.

DNA capture rate measurements were performed separately from phi29 DNAP—DNA translocation experiments. After determining the unblocked pore current, as explained above, instances of DNA capture were found using a thresholding method with unique current cutoff values for each salt condition. For each asymmetric condition, DNA capture rate was determined by dividing the number of instances of DNA capture by the total duration of unblocked pore time.

## Results

### Influence of *cis* [Cl^-^] on ion currents through MspA

**Fig 3:**
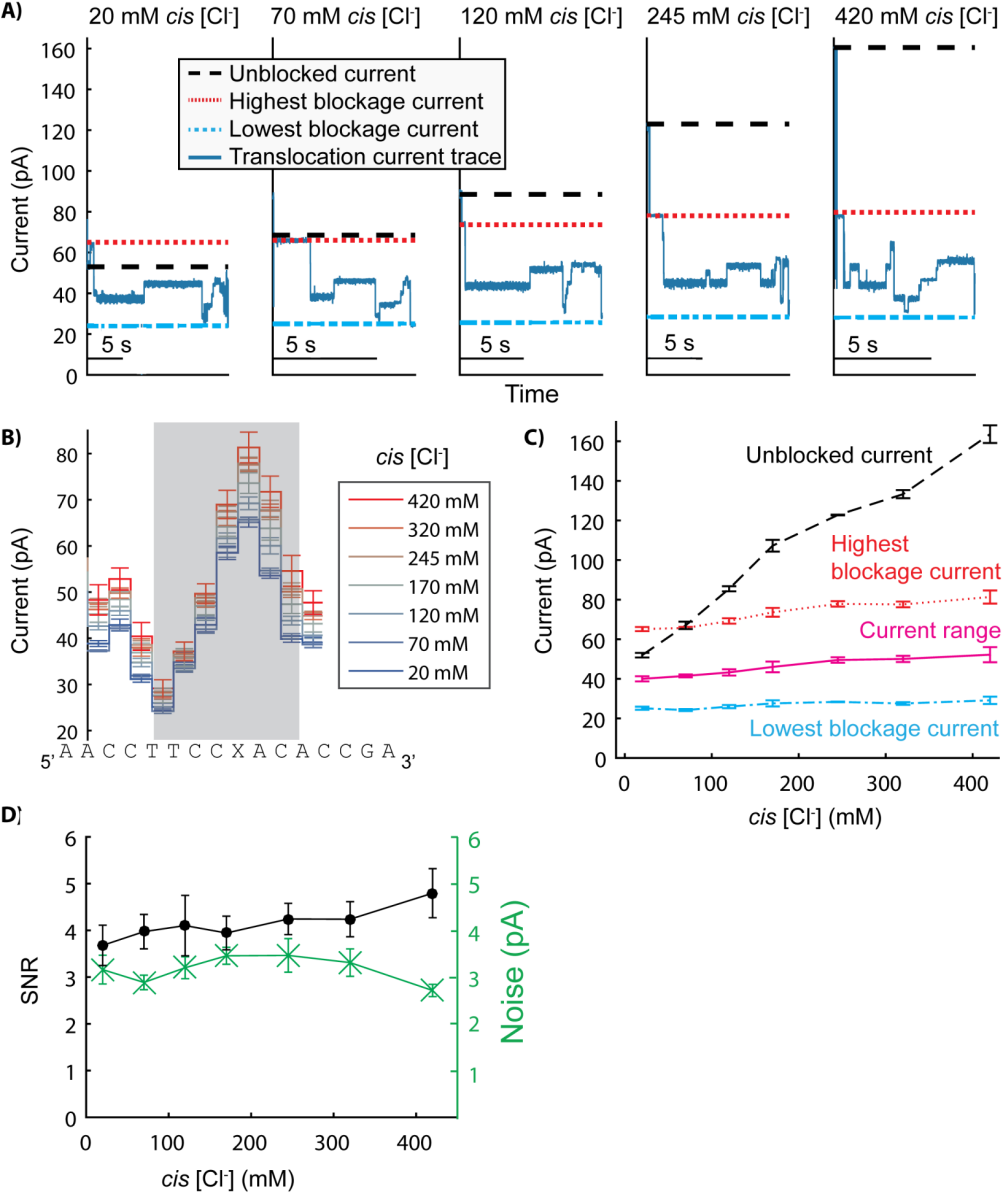
Effect of *cis* [Cl^-^] on ion currents through MspA. (A) Current traces of phi29 DNAP controlled DNA translocation were recorded over a range of *cis* [Cl^-^] and at 500 mM *trans* [K^+^]. All experiments were performed at pH 8.00 ± 0.05. Panel A shows individual reads of the same DNA sequence over a range of concentrations (20 mM to 420 mM *cis* [Cl^-^] with an applied voltage of 180 mV). For each *cis* condition, the unblocked pore current (black dashed line) was calculated, as well as the highest current level (red dotted line) and lowest current level (cyan dotted line) using the consensus current traces from panel B. (B) At least 8 reads at each *cis* condition were used to generate a consensus current trace for the translocation of the DNA sequence in each condition. Panel B shows an 11 nucleotide section of the consensus plots for each condition. The 7 current levels shaded in grey were used for the SNR and noise analysis in panel D. Errors in current are S.E. The DNA sequence plotted underneath each current level correspond to the nucleotides in the constriction of MspA during that state. (C) Highest current level, lowest current level, and the range of current blockages were calculated for each *cis* condition using the consensus current traces from panel B. Errors are S.E. (D) Average noise and signal to noise ratio (SNR) were calculated for each *cis* concentration using only the level transitions in the region shaded in grey in panel B. Errors in average noise and SNR are S.E.M. (standard error of the mean).

With an applied voltage of 180 mV, we varied *cis* [KCl] from 0 mM to 400 mM with a fixed *trans* [KCl] of 500 mM. We recorded unblocked pore current as well as many instances of phi29 DNAP controlled DNA translocation (n = 8 to 43) at each condition (Fig 3). *Cis* [Cl^-^] ([KCl] + 20 mM) is indicated as the applied electric field direction drives Cl^-^ ions from *cis* to *trans*. The unblocked MspA current (black dashed line in Fig 3A and Fig 3C) exhibits an approximately linear response to *cis* [Cl^-^] at 500 mM *trans* KCl. Although the open pore current is significantly reduced at low *cis* [Cl^-^] (51.9 ± 1.2 pA at 20 mM *cis* [Cl^-^]), single MspA pores are still identifiable.

Fig 3A displays example current traces of phi29 DNAP controlling DNA translocation of the same DNA sequence in five separate *cis* conditions at 500 mM *trans* KCl. Consensus current traces (Fig 3B), generated from the set of translocation reads at each *cis* condition, demonstrate that the current pattern for the DNA sequence is consistent across salt concentrations even though the magnitude of each current level varies between conditions. For the DNA sequence analyzed here, the highest current level during DNA translocation involves an abasic site denoted by X. Both the highest (red dotted line in Fig 3A and 3C) and lowest (cyan dotted line in Fig 3A and 3C) ioncurrent levels, averaged over at least 8 events, increase with *cis* [Cl^-^]. The current range, the difference between the highest current level and the lowest current level, is a good metric to assess the signal magnitude at each asymmetric salt profile (magenta curve Fig 3C). The current range during DNA translocation increases only slightly with *cis* [Cl^-^] (Fig 3C). Between 20 mM and 420 mM *cis* [Cl^-^], the current range only increases by ~ 30% while the open state current nearly doubles. At ~70 mM *cis* [Cl^-^], the highest current during DNA blockage is equal to the open pore current. Below 70 mM *cis* [Cl^-^], the highest current level is larger than the unblocked pore current. Specifically, the highest current is 13.2 ± 1.6 pA greater than the open state at 20 mM *cis* [Cl^-^].

We next investigated the effect of *cis* [Cl^-^] on noise of translocation currents. For every DNA translocation event, we calculated the standard error for each current level between the lowest and highest currents (region shaded in grey in Fig 3B). The green stars in Fig 3D show the average for every noise measurement over the range of *cis* [Cl^-^]. Average current level noise is not statistically significantly correlated with *cis* [Cl^-^]. Between 20 mM and 420 mM [Cl^-^], the average noise remains relatively unchanged. With both signal and noise measurements available, we calculated the signal-to-noise ratio (SNR) for each *cis* [Cl^-^] using a t-test (Fig 3D). For every *cis* [Cl^-^] event, we calculated the average t-test value for the current transitions during DNA translocation (again focusing on the shaded in grey in Fig 3B). The average SNR, plotted as the black circles in Fig 3D, follows a similar trend to the current signal range between 20 mM and 420 mM *cis* [Cl^-^], increasing from 3.7 ± 0.4 to 4.8 ± 0.5 (~ 30% increase). Decreased functionality of phi29 DNAP at high ionic concentrations prohibited experiments at *cis* [Cl^-^] above this range.

### Influence of *trans* [K^+^] on ion currents through MspA

**Fig 4:**
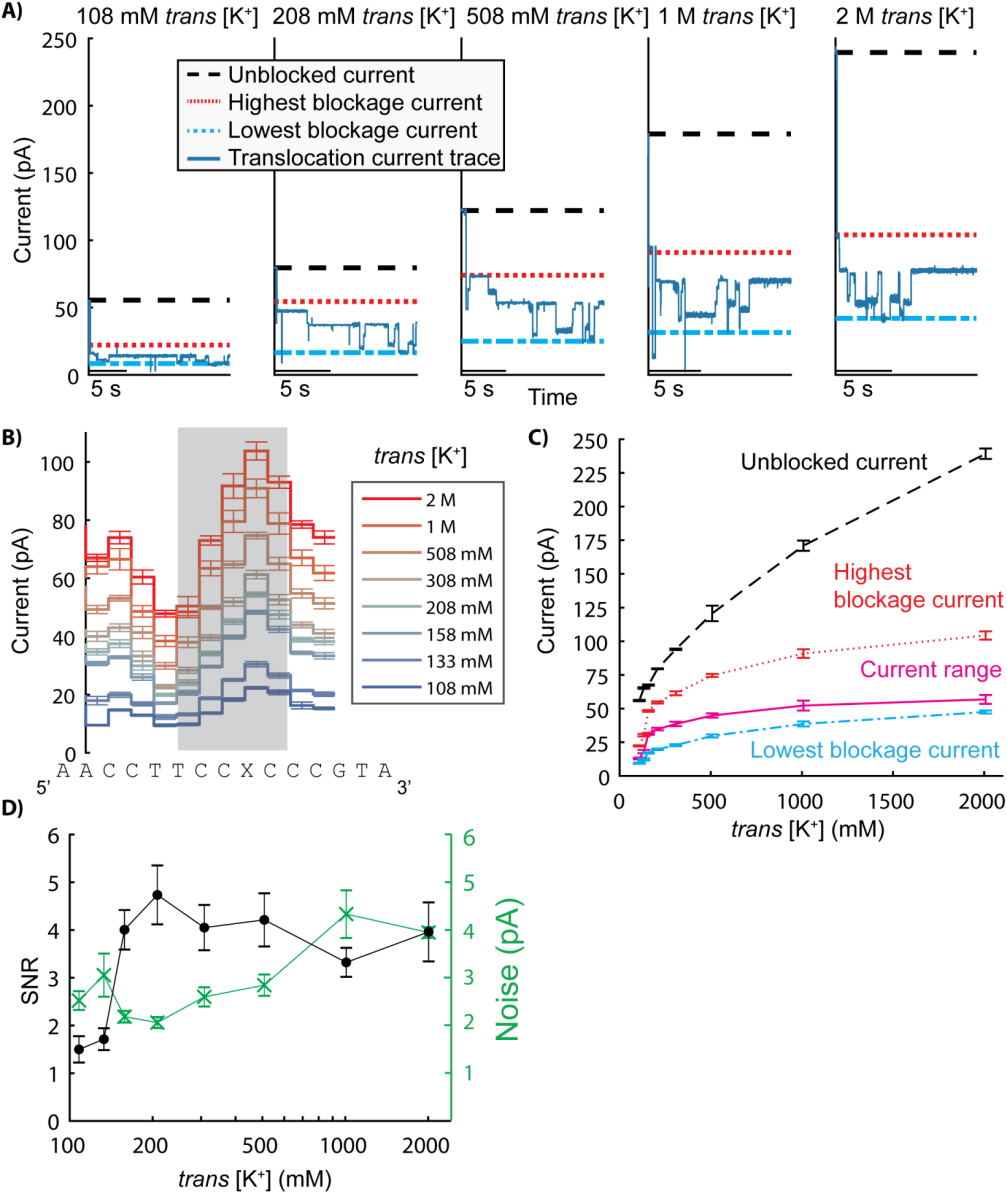
Effect of *trans* [K^+^] on currents through MspA. (A) Current traces of phi29 DNAP controlled DNA translocation were recorded over a range of *trans* [K^+^] and at 200 mM *cis* [Cl^-^]. All experiments were performed at pH 8.00 ± 0.05. Panel A shows individual reads of the same DNA sequence over a range of concentrations (108 mM to 2 M *trans* [K^+^] with an applied voltage of 180 mV). For each *trans* condition, the unblocked pore current (black dashed line) was calculated, as well as the highest blockage current (red dotted line) and lowest blockage current (cyan dotted line) using the consensus current traces from panel B. (B) Multiple reads at each *trans* condition were used to generate a consensus current trace for the translocation of the DNA sequence in each condition. Panel B shows an 11 nucleotide section of the consensus plots for each condition. The DNA sequence plotted underneath each current level correspond to the nucleotides in the constriction of MspA during that state. The 5 current levels shaded in grey were used for the SNR and noise analysis in panel D. Errors are S.E. (C) Highest current blockage, lowest current blockage, and the range of current blockages were calculated for each *trans* condition using the consensus current traces from panel B. Errors are S.E. (D) Average noise and signal to noise ratio (SNR) were calculated for each *cis* concentration using only the level transitions in the region shaded in grey in panel B. The x-axis is logarithmic. Errors are S.E.M.

We next varied *trans* [KCl] from 100 mM to 2 M while keeping the *cis* concentration of KCl at 200 mM with an applied voltage of 180 mV (Fig 4). We used a slightly different DNA sequence (S1 Table) and gathered many phi29 DNAP controlled DNA translocation reads at each condition (N = 16 to 76). *Trans* [K^+^] ([KCl] + 8 mM) is indicated, as K^+^ is responsible for generating current from the *trans* chamber as the applied electric field direction drives K^+^ ions from *trans* to *cis*. Unblocked pore currents (black dashed line in Fig 4A and Fig 4C) vary significantly with *trans* [K^+^] at 200 mM *cis* KCl, although the relationship differs from the linear response seen with varying [*cis*] (Fig 3C vs Fig 4C). The DNA translocation current range drops off sharply at low *trans* [K^+^]. Between 108 mM and 158 mM *trans* [K^+^], the current range increases by almost 150% (12.9 ± 0.6 pA to 31.3 ± 0.8 pA). Small variations of *trans* [K^+^] produce large changes in signal over this range. However, as *trans* [K^+^] is increased, the magnitude of change in current range is diminished, exhibiting a plateau-like effect. Between 158 mM and 508 mM, the current range still increases by ~ 40% (from 31.3 ± 0.8 pA to 44.9 ± 1.6 pA), but further increasing *trans* [K+] to 2 M only changes the current range by ~ 25% (from 44.9 ± 1.6 pA to 56.9 ± 3.3 pA).

We analyzed noise and SNR over the range of *trans* [K^+^] concentrations at 200 mM *cis* KCl, focusing on the level transitions in the region shaded in grey in Fig 4B. Average DNA translocation current noise (green stars in Fig 4D) varies more significantly for increasing *trans* [K^+^] than for increasing *cis* [Cl^-^] (Fig 3D vs Fig 4D). While current range changes dramatically between 100 mM and 200 mM *trans* [K^+^] (Fig 3C), the majority of the noise increase occurs above this range, between 200 mM and 1 M *trans* [K^+^]. Correspondingly, the average SNR, plotted as the black circles in Fig 4D, rises sharply between 108 mM and 158 mM, increasing by a factor of ~3 (1.5 ± 0.3 to 4.7 ± 0.6). As *trans* [K^+^] is further increased, the SNR begins to decrease due to the increased noise and plateauing signal magnitude. At a fixed *cis* [KCl] of 200 mM, increasing the *trans* [K^+^] above 200 mM provides little benefit in distinguishing current transitions.

### Asymmetric salt profiles affect DNA capture rate

**Fig 5:**
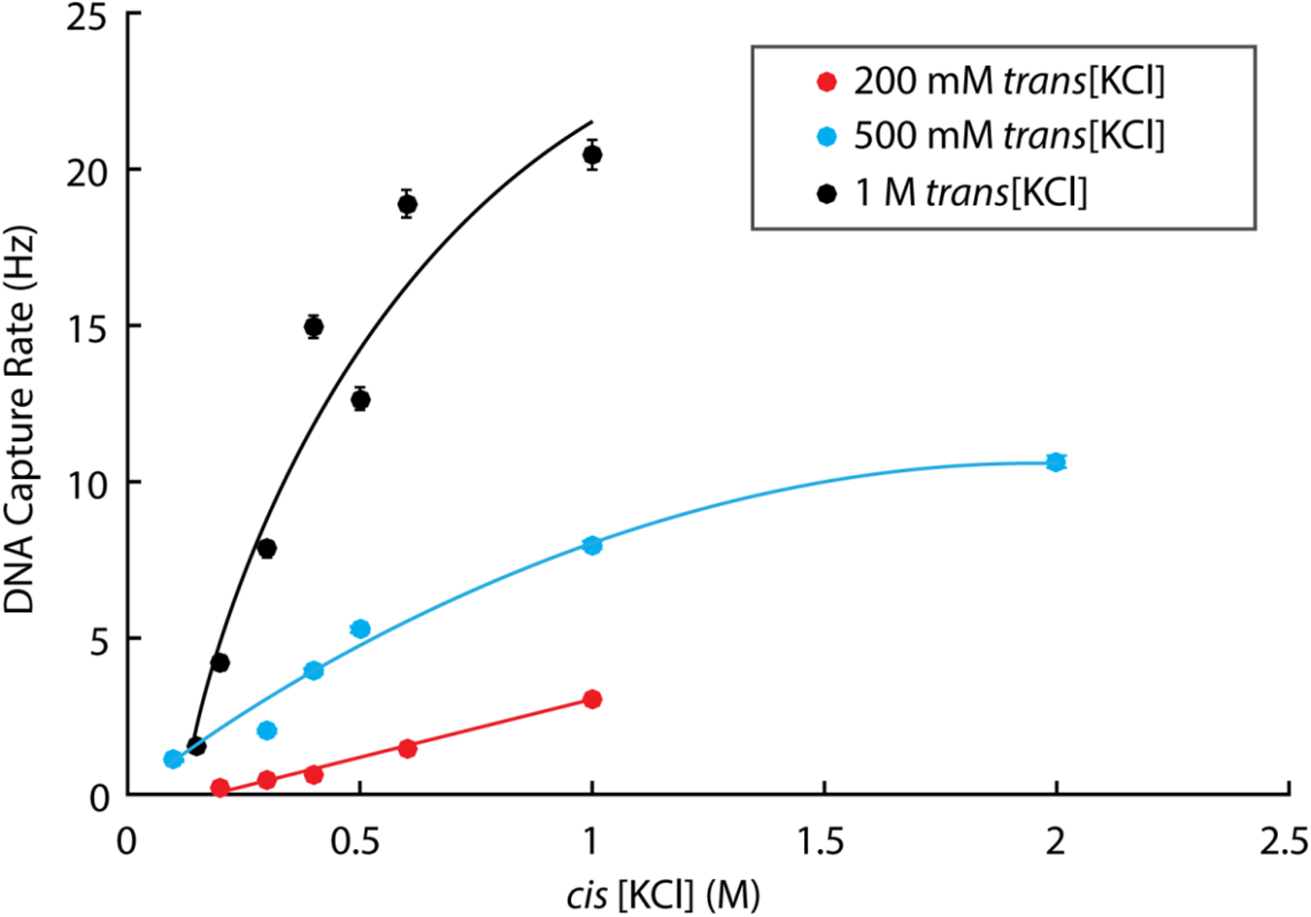
Effect of *cis* and *trans* [KCl] on DNA capture rate by MspA. DNA capture rate, the number of DNA molecules threading through MspA per second, was measured using short hairpin DNA (500 nM) over a range of *cis* [KCl] at three *trans* [KCl] with an applied voltage of 180 mV. No phi29 DNAP enzyme was included in this set of experiments. Trend lines are to guide the eye. Errors are S.E.M.

Salt concentration influences experimental throughput in addition to SNR in nanopore sequencing. We measured the effect of asymmetric salt profiles on the rate at which DNA molecules are captured into MspA with an applied voltage of 180 mV at pH 8.00 ± 0.05 (DNA Capture Rate, Fig 5). At three different *trans* concentrations (200 mM, 500 mM, and 1 M [KCl]), capture rate of free DNA (no enzyme present) increases with *cis* [KCl]. For each *cis* concentration in which multiple *trans* concentrations were sampled, capture rate also increases with *trans* [KCl].

## Discussion

### Enhanced K^+^ selectivity during DNA translocation

This study helps uncover the individual contribution of *cis* and *trans* ions to the ion current through MspA, revealing that the presence of negatively charged DNA in the pore promotes the transit of cations from *trans* to *cis* and minimizes the transit of anions from *cis* to *trans*. While the applied electric field controls the direction of ion flow (positive ions (K^+^) from *trans* to *cis* and negative ions (Cl^-^) from *cis* to *trans*), the rate of ion passage in each direction is also dependent on the microenvironment within the pore that the ions must pass through. The MspA M2NNN mutant used in this study is neutral within the pore constriction, with neutral asparagines replacing negatively charged aspartic acid residues in the wild type [4]. During DNA translocation, the constriction environment changes both physically and electrostatically. Along with the DNA bases occluding the pore constriction, each phosphate along the backbone of the DNA introduces a negative charge into the pore. By comparing the ion current through MspA with and without DNA (blocked pore current and open state current Figs 3 and 4) in identical ion concentrations and applied voltage, the overall effect of this altered microenvironment on ion passage becomes apparent. In all of the asymmetric salt profiles sampled (Figs 3 and 4), except below ~70 mM *cis* at 500 mM *trans* [KCl], the open state current is larger than the highest DNA blockage current. The combined rates of Cl^-^ flow from *cis* to *trans* and K^+^ flow from *trans* to *cis* are reduced by the presence of DNA within the pore.

Assessing the relationship between ion current signal and *cis* and *trans* ion concentration independently can untangle the specific contribution of each ion type to the overall ion current. The relationship between ion current and *cis* [Cl^-^] (Fig 3C) demonstrates that the presence of DNA diminishes the ion current sensitivity to *cis* [Cl^-^]. As *cis* [Cl^-^] varies from 20 mM to 420 mM with a constant *trans* [KCl] of 500 mM, the currents through MspA with DNA present in the pore (highest and lowest blockage currents) increase at a much slower rate over the entire range than without DNA (open state current). However, with minimal Cl^-^ present to flow from *cis* to *trans (*below ~70 mM *cis* Cl^-^), the highest DNA blockage current actually exceeds the open pore current, indicating that while Cl^-^ flow from *cis* to *trans* is minimized by the presence of DNA within the pore, K^+^ flow from *trans* to *cis* is enhanced. The DNA sequence leading to the highest current level in this strand contains an abasic residue, consisting of a missing base and only DNA phosphate sugar backbone, further suggesting that the negative charge promotes cation passage instead of some DNA-nucleobase-specific interaction with the cations.

The relationship between *trans* [K^+^] and DNA blockage current is consistent with this model of ion flow (Fig 4). Below ~200 mM *trans* K^+^ at 200 mM *cis* KCl, the DNA blockage currents drop off steeply (Fig 4C) to the point at which distinguishing adjacent current translocation steps becomes prohibitively difficult below 100 mM *trans* K^+^ (Fig 4B and Fig 4D). This extreme sensitivity to *trans* [K^+^] lessens as *trans* [K^+^] is increased above 200 mM, and the DNA blockage currents begin to saturate. Current saturation is consistent with other biological porins that demonstrate large selectivity bias (preferring transit of one ion species over another) due to charged residues [15, 16]. In these previous studies, the proposed model suggests that each negative charge within the pore represents a binding site for a positive ion during transit. At a high positive ion concentration, all of these ion binding sites are always occupied and mask the negative charges, which discourages further K^+^ ions moving from *trans* to *cis*. The limiting rate for ion transfer is no longer the time it takes to fill an evacuated site, but instead the time it takes for the positive ions to move from one site to the next. At this point, the flow of ions is independent of ion concentration and depends only on the overall membrane potential or applied voltage.

Overall, these phenomena suggest a complex model of ion flow for DNA translocation currents. When DNA enters the pore, the cationic selectivity of MspA increases as the charged backbone actually promotes K^+^ flow from *trans* to *cis* and minimizes Cl^-^ flow from *cis* to *trans*. The specific nucleobases further modulate and block this flow depending on their type as the motor enzyme pulls DNA through the pore.

### Benefits of asymmetric salt profiles for nanopore sequencing

Salt concentration affects many experimental parameters in nanopore sequencing, including signal-to-noise ratio (SNR) during DNA translocation, motor enzyme function, and DNA capture rate. Using the results presented in this study, *cis* and *trans* conditions can be tuned independently to balance and optimize these parameters. Increasing SNR improves resolution when monitoring DNA translocation and could lead to improved sequencing accuracies. Due to the sharp dropoff in SNR at low *trans* [K^+^] (Fig 4D), resolving DNA translocation becomes difficult at *trans* concentrations below 150 mM. Additionally, increasing *trans* [K^+^] above 200 mM does not improve SNR, suggesting an ideal *trans* [K^+^] of above ~150 mM to 200 mM with an applied voltage of 180 mV. Increasing *cis* [Cl^-^] also marginally improves SNR (Fig 3D), although even at negligible *cis* [Cl^-^], as long as *trans* [K^+^] is maintained, the SNR is not prohibitive to monitoring DNA translocation.

Simultaneously, altering the *cis* well conditions can affect the activity of the motor enzyme controlling DNA translocation in nanopore experiments. Salt concentration can affect the stepping behaviour, processivity, and binding affinity of motor enzymes [17, 18]. Even with reasonable SNR, enzymatic “missteps” along DNA (backtracking, skipping, or toggling between translocation states) obfuscate analysis and contribute to errors in nanopore DNA sequencing. Because the motor enzyme is only present in the *cis* chamber and not *trans*, by tuning the *cis* salt concentration independently to the preferred operating conditions of the motor enzyme, enzymatic “missteps” may be minimized. Phi29 DNAP, the motor enzyme used in this study, can operate over a wide range of salt concentrations, enabling nanopore DNA translocation experiments with negligible *cis* [KCl] and up to 400 mM KCl (Fig 3). However, many motor enzymes that may be useful for nanopore sequencing (polymerases, helicases and recombinases) function only in a narrower range of salt concentrations, and many lose binding affinity above 50–100 mM (19,20). By maintaining the *trans* concentration for SNR, and adjusting the *cis* concentration specifically for enzymatic function, salt sensitive motor enzymes can be used for nanopore sequencing.

Additionally, both *cis* and *trans* concentrations can be altered to affect experimental throughput. In experiments with solid-state nanopores, Wanunu et al. demonstrated that asymmetric salt profiles with [*trans*] > [*cis*] enhance the electric field in *cis* and subsequently increase DNA capture rate [11, 19]. While this same phenomena has also been observed in biological nanopores, a separate effect simultaneously influences DNA capture rate with changing *cis* concentration due to the electrostatic interactions between a biological pore and DNA. Specifically, Jeon et al. determined that increasing *cis* ion concentration shields the electrostatic interactions between the biological pore α-hemolysin and a negatively charged polymer [9]. With α-hemolysin at pH 7.5, these interactions are repulsive, and, therefore, increased ionic shielding promotes polymer capture. In our experiments with MspA, with three separate *trans* KCl concentrations, we see an increased DNA capture rate as *cis* [KCl] is increased at pH 8 (Fig 5). Although MspA mutant M2NNN is neutral within the constriction, the rim of the pore is net-negatively charged, creating an electrostatic barrier for DNA capture similar to α-hemolysin at the same pH. While the benefit of increasing *cis* KCl concentration is apparent at all of the asymmetric salt profiles tested, increasing *trans* concentration at a given *cis* concentration also promotes DNA capture, suggesting that the ratio of *trans* to *cis* concentration still influences DNA capture with MspA.

When choosing *cis* and *trans* concentrations for nanopore sequencing with MspA, optimizing DNA capture rate can decrease the requisite amount of initial reagents (DNA, enzymes) to maintain experimental throughput. As described above, *cis* concentrations are limited by the operating conditions of the motor enzyme controlling DNA translocation. However, even though the SNR benefits of increasing *trans* concentration are negligible above ~150–200 mM (Fig 4D), *trans* concentrations can still be maximized to increase DNA capture rate.

In summary, using asymmetric salt profiles, i.e. tuning *cis* and *trans* salt concentrations independently, allows simultaneous optimization of multiple parameters for nanopore DNA sequencing with MspA. *Cis* concentration can match the preferred operating conditions of the motor enzyme controlling DNA translocation, even for salt-sensitive motor enzymes, with minimal effect to SNR. High *trans* concentration optimizes SNR and experimental throughput. By following these guidelines, new DNA motor enzymes can be effectively tested for nanopore DNA sequencing with MspA. Furthermore, in addition to DNA sequencing applications, the data gathered with each new DNA motor enzyme provides valuable scientific insight into enzyme function. By monitoring the controlled translocation of DNA through MspA by an enzyme, precise information about the kinetics and stepping behavior of that enzyme become available. In fact, this technique, Single-molecule Picometer Resolution Nanopore Tweezers (or SPRNT) [7], permits an order of magnitude improvement in spatiotemporal resolution over optical tweezers, a standard technology for single-molecule studies of nucleic acid processing enzymes. Using asymmetric salt profiles, SPRNT reaction conditions can also be optimized in the *cis* compartment, while maintaining high *trans* [K^+^] to obtain a high SNR. This study provides a guide for studying DNA motor enzymes at unprecedented resolution regardless of the enzymes’ preferred operating conditions.

## Supporting information

S1 Fig DNA constructs. Schematics of the DNA constructs used in the DNA translocation experiments (A) and DNA capture rate experiments (B).
(TIF)

S2 Fig Effect of *cis* and *trans* [KCl] ratio on DNA capture rate. DNA capture rate, the number of DNA molecules threading through MspA per second, was measured using short hairpin DNA (500 nM) over a range of *cis* [KCl] at three *trans* [KCl] with an applied voltage of 180 mV. No phi29 DNAP enzyme was included in this set of experiments. Errors are S.E.M.
(TIF)

S1 Table DNA sequences and experimental statistics. A list of all DNA strands and complements used in this study, and the number of pores and events used in the creation of the consensus sequences.
(PNG)
