# Supplementary figures and images for: Investigating asymmetric salt profiles for nanopore DNA sequencing with biological porin MspA
Source: PLoS One. 2017 Jul 27;12(7):e0181599. doi: 10.1371/journal.pone.0181599 (PMC5531483; doi:10.1371/journal.pone.0181599)

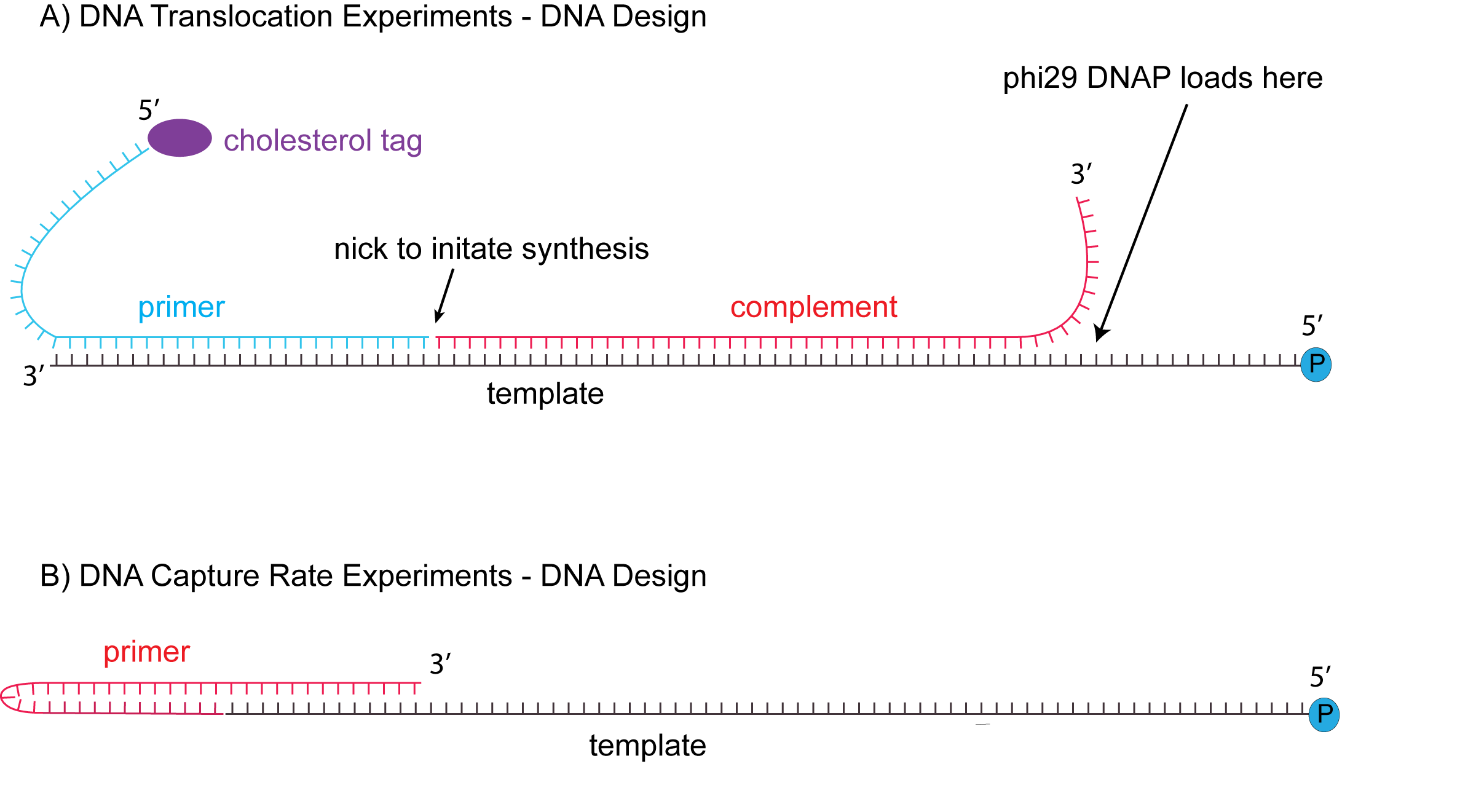

Supplement: S1 Fig — Schematics of the DNA constructs used in the DNA translocation experiments (A) and DNA capture rate experiments (B). (TIF) [file pone.0181599.s001.tif]

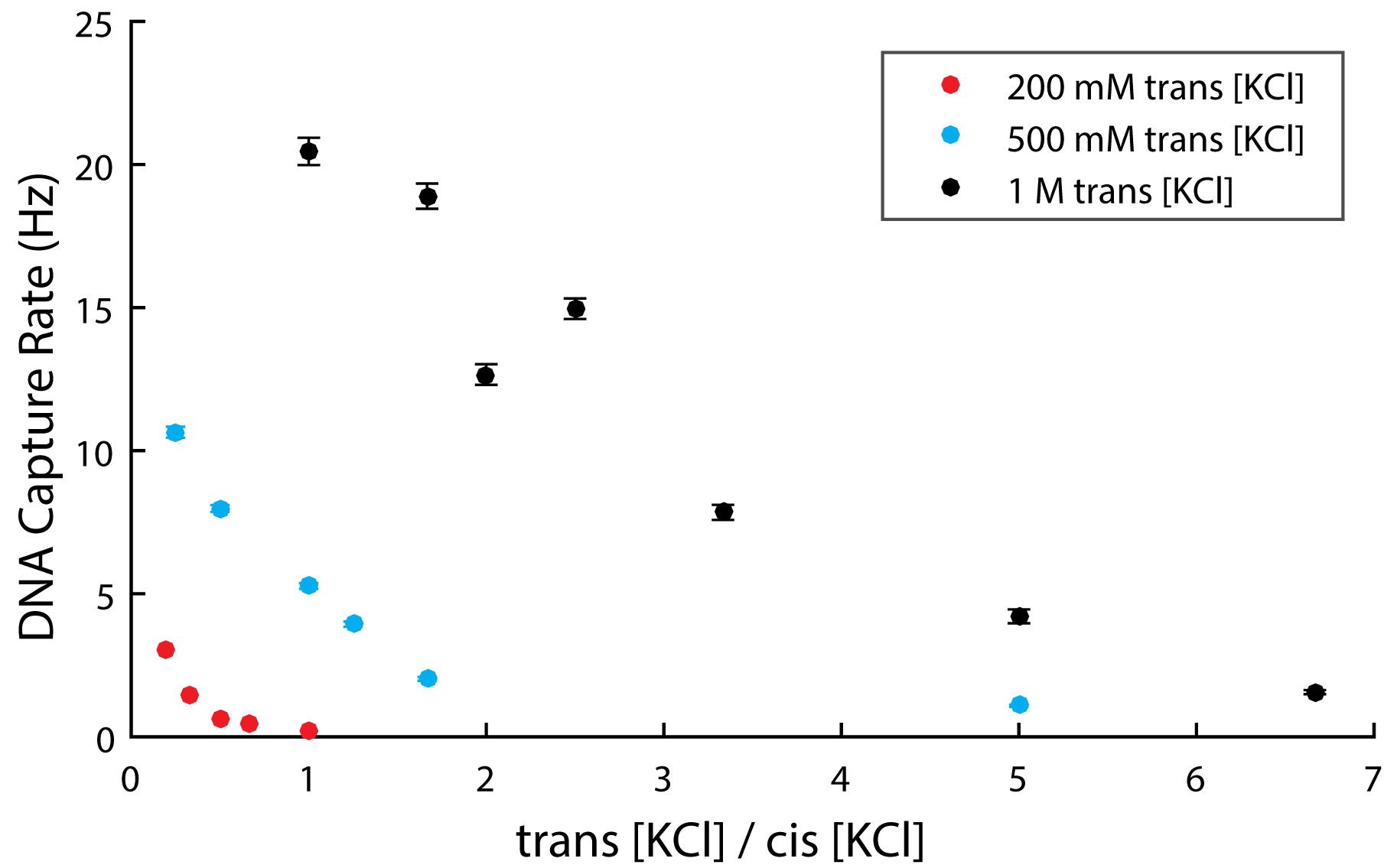

Supplement: S2 Fig — DNA capture rate, the number of DNA molecules threading through MspA per second, was measured using short hairpin DNA (500 nM) over a range of cis [KCl] at three trans [KCl] with an applied voltage of 180 mV. No phi29 DNAP enzyme was included in this set of experiments. Errors are S.E.M. (TIF) [file pone.0181599.s002.tif]

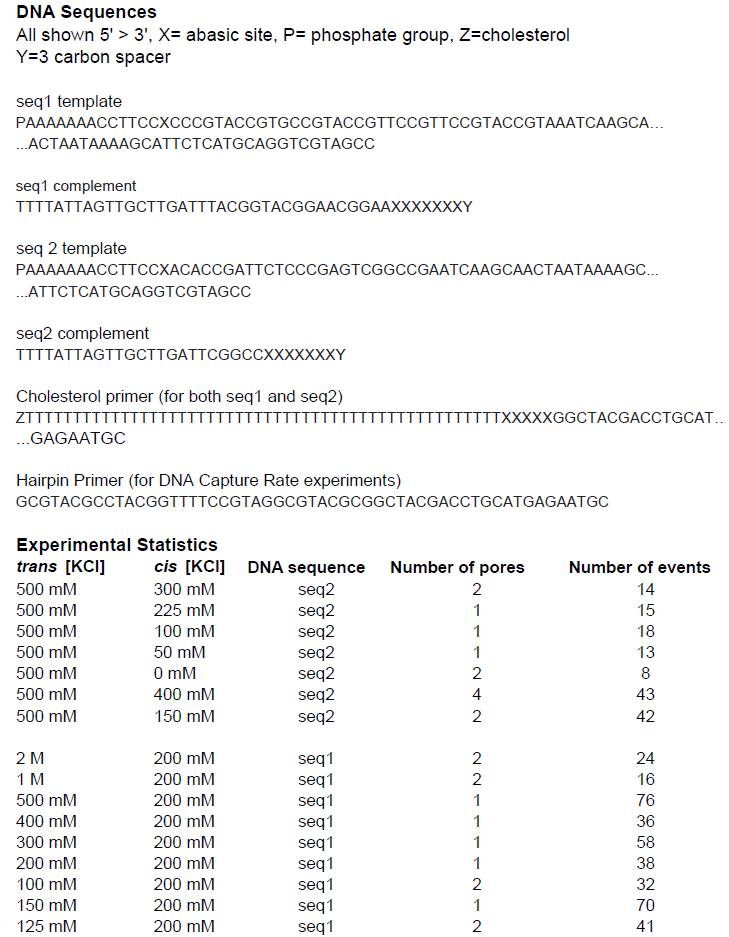

Supplement: S1 Table — A list of all DNA strands and complements used in this study, and the number of pores and events used in the creation of the consensus sequences. (PNG) [file pone.0181599.s003.PNG]
